# Snake and Bird Predation Drive the Repeated Convergent Evolution of Correlated Life History Traits and Phenotype in the Izu Island Scincid Lizard (*Plestiodon latiscutatus*)

**DOI:** 10.1371/journal.pone.0092233

**Published:** 2014-03-25

**Authors:** Matthew C. Brandley, Takeo Kuriyama, Masami Hasegawa

**Affiliations:** 1 School of Biological Sciences, University of Sydney, Sydney, NSW, Australia; 2 Faculty of Science, Toho University, Funabashi City, Chiba, Japan; Field Museum of Natural History, United States of America

## Abstract

Predation may create strong natural selection pressure on the phenotype and life history characteristics of prey species. The Izu scincid lizards (*Plestiodon latiscutatus*) that inhabit the four Japanese Izu Islands with only bird predators are drab brown, mature later, lay small clutches of large eggs, and hatch large neonates. In contrast, skinks on seven islands with both snake and bird predators are conspicuously colored, mature early, lay large clutches of small eggs, and hatch small neonates. We test the hypothesis that these suites of traits have evolved independently on each island via natural selection pressures from one of two predator regimes – birds-only and birds + snakes. Using two mtDNA genes and a nuclear locus, we infer a time-calibrated phylogeny of *P. latiscutatus* that reveals a basal split between Mikura and all islands south, and Miyake, all islands north, and the Izu Peninsula. Populations inhabiting Miyake, Niijima, Shikine, and Toshima are not monophyletic, suggesting either multiple colonizations or an artifact of incomplete lineage sorting (ILS). We therefore developed novel phylogenetic comparative analyses that assume either a multiple colonization or more restrictive single colonization ILS scenario and found 1) statistically significant support for the of different suites of phenotypic and life history characteristics with the presence of bird-only or bird + snake predator assemblages, and 2) strong phylogenetic support for at least two independent derivations of either the “bird-only” or “snakes + birds” phenotypes regardless of colonization scenario. Finally, our time-calibrated phylogeographic analysis supports the conclusion that the ancestor to modern Izu Island *P. latiscutatus* dispersed from the mainland to the Izu proto-islands between 3–7.6 million years ago (Ma). These lineages remained present in the area during successive formation of the islands, with one lineage re-colonizing the mainland 0.24-0.7 Ma.

## Introduction

Predation may result in strong selection on prey phenotype and life history. For example, predation by visually-orienting predators may impose a strong natural selection pressure to evolve a cryptic color pattern in prey species, or life history traits that improve predator avoidance [1]. Extensive studies of poeciliid fish have demonstrated an association between high rates of predation and a maternal life history strategy that favors early sexual maturity and high fecundity (i.e., clutch size) at the expense of offspring size (e.g., [2]–[5]). That this life history strategy has evolved multiple times both within and amongst species in high predation environments is strong evidence that this life history strategy is a response to natural selection due to predation.

Detecting this convergent evolution of life history requires knowledge of a species’ evolutionary and/or biogeographical history. For example, if two populations share a unique phenotype, but genetic data reveals they are also sister lineages, we could conclude that the phenotype evolved once prior to the splitting of the lineage. Similarly, if isolated habitats with no connectivity are independently colonized by the same species, these populations each become a “natural experiment” to test how species evolve in response to different selection pressures.

**Figure 1:**
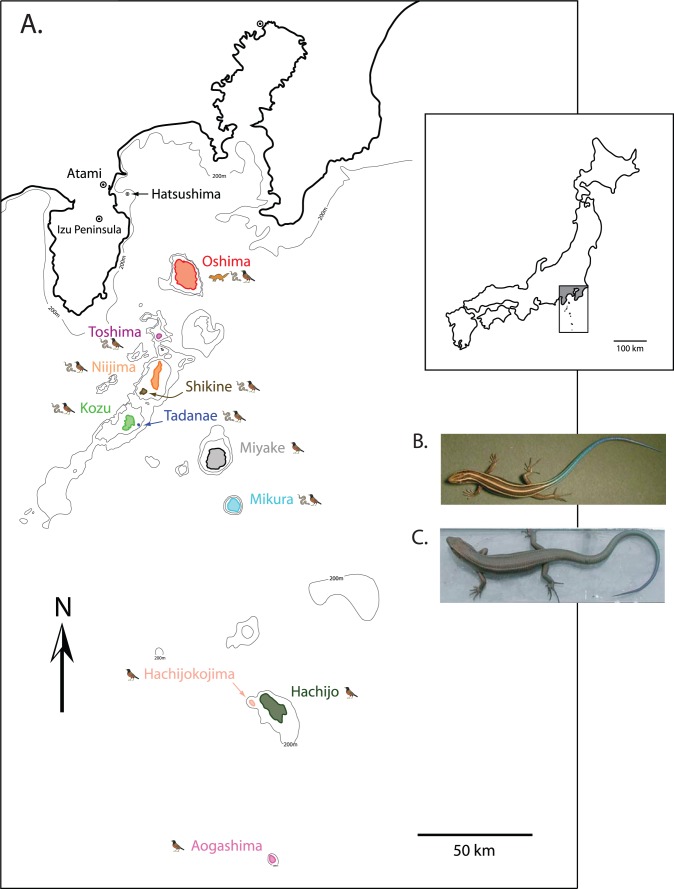
A. Map of the Izu Islands and nearby mainland Japan. The weasel (*Mustela itatsi*), snake (*Elaphe quadrivirgata*), and bird (*Turdus celaenops*) icons indicate whether those predators historically inhabit the island (the weasel has been subsequently introduced to Toshima, Miyake, Hachijojima and Aogashima since the 1930s). Interior bathymetric lines indicate a depth of 100 m and exterior lines a depth of 200 m unless otherwise specified; B. typical striped morph of *Plestiodon latiscutatus* inhabiting islands with snake predators (Oshima, Toshima, Niijima, Shikine, Kozu, Tadanae, and Mikura); C. typical drab morph of the same species inhabiting bird-only predator islands (Miyake, Hachijojima, Hachijokojima, and Aogashima).

Because oceanic islands form with no connection to the mainland and are colonized only by limited dispersal, they serve as ‘blank slates’ on which we can observe the varied outcome of ecological and evolutionary processes. One such oceanic island model system is the Japanese Izu Islands (Fig. 1). The Izu Islands are an ideal study system because of their geologic and taxonomic diversity, range in island size (approximately 10 to 9908 ha), and distance of islands from the mainland (∼23 to ∼260 km). In particular, the skink *Plestiodon latiscutatus* (formerly *Eumeces okadae* [6]) inhabits all Izu Islands, yet one or more of its major predators the Japanese weasel (*Mustela itatsi*), Izu Island Thrush (*Turdus celaenops*), and four-lined ratsnake (*Elaphe quadrivirgata*) inhabits every island (see Table 1). For example, *M. itatsi* is native only to Oshima (although it was subsequently introduced to, and persists in, Toshima, Miyake, Hachijojima and Aogashima [8]–[10]); at least one species of predatory bird (especially *T. celaenops*) inhabits all the major Izu Islands [11], [12]; ratsnakes inhabit most, but not all of the islands ([12]; Fig. 1). The islands’ similarity in climate and vegetation makes them a unique test case to evaluate the effects of different predator assemblages on the evolution of life history of prey species. Moreover, as previous phylogeographic research has revealed varied patterns of historical colonization of the Izu Islands in *Campanula* plants [13], [14], *Euhadra* snails [15], *Apodemus* mice [16]–[18], and the four-lined ratsnake, *Elaphe quadrivirgata* [19], it is likely that the order in which these islands were colonized would also substantially affect subsequent ecological and phenotypic evolution of the species community (e.g., whether the colonizer was from a distant, mainland-adapted or closer, island-adapted population).

**Table 1:** Predator assemblages of islands in this study with summary statistics of life history and phenotype traits of each sampled Izu Island population of *Plestiodon latiscutatus*.

		Hatchlings								Mothers							
		SVL (mm)		Mass (g)		Stripe index		Proportion of blue tail		SVL (mm)		Clutch size (eggs)			Egg mass (g)		
Population	Predator	Mean±SD	N	Mean±SD	N	Mean±SD	N	Mean±SD	N	Mean±SD	N		Mean±SD	N	Mean±SD	N	
Aogashima	Birds	30.4±1.3	68	0.57±0.08	69	3.9±2.6	69	0.38±0.03	37	85.3±4.6	20		6.5±1.9	20	0.057±0.007	11	
Hachijokojima	Birds	31.4±1.5	107	0.61±0.09	108	7.3±2.6	108	0.37±0.04	108	84.0±3.8	35		6.6±1.9	35	0.062±0.007	35	
Kozu	Birds + snakes	28.8±1.2	142	0.49±0.06	143	16.2±1.2	143	0.51±0.03	70	80.2±4.7	46		7.8±1.7	46	0.048±0.005	36	
Mikura	Birds + snakes	30.5±1.0	61	0.55±0.05	62	10.3±2.4	62	0.49±0.09	56	80.0±4.0	49		7.1±1.8	52	0.051±0.006	35	
Miyake	Birds	30.6±1.1	73	0.63±0.07	42	3.4±3.1	74	0.49±0.03	46	82.3±4.3	92		7.3±1.9	115	0.059±0.007	37	
Niijima	Birds + snakes	29.2±1.5	141	0.51±0.07	120	15.0±1.2	142	0.49±0.03	132	82.8±4.7	37		8.2±2.3	38	0.052±0.007	19	
Oshima	Mammals + birds + snakes	26.5±1.1	175	0.40±0.06	158	16.1±0.8	176	0.55±0.04	137	78.4±6.2	30		8.8±2.0	28	0.041±0.005	20	
Shikine	Birds + snakes	29.2±1.0	70	0.52±0.04	71	11.0±3.4	71	0.55±0.03	30	80.1±3.5	23		8.0±1.0	22	0.051±0.006	20	
Toshima	Birds + snakes	27.5±1.1	120	0.42±0.05	118	16.1±1.1	121	0.52±0.03	75	81.2±4.2	26		9.0±2.1	26	0.043±0.006	20	

Previous research has identified suites of life history traits in the skink *P. latiscutatus* that appear to correlate with each island’s predator assemblage [8], [9], [12], [20]–[22]. For example, skinks on Oshima (the largest island that is nearest to the mainland and with the most taxonomically diverse assemblage of predators) mature earlier with a small body and lay comparatively large clutches of small eggs [12]. Whereas skinks that inhabit bird-only islands mature later with a large body size and lay smaller clutches with larger eggs [12]. Moreover, there are striking differences in color pattern between *P. latiscutatus* populations that inhabit snake + bird and bird-only predator islands. Skinks on snake + bird islands have a dorsal color pattern with vivid yellow stripes and pronounced blue tail in juveniles ([24]; Fig. 1B), whereas skink populations on bird-only islands are uniformly drab brown in colour (Fig. 1C). Given the similar habitats amongst the islands, these results suggest a causal link between the presence of particular predators and the life history and color pattern evolution of their skink prey.

Evidence that these phenotype and life history traits evolved independently on two more islands with the identical suites of predators would indicate that predator diversity was the primary natural selection pressure. However, because phylogenetic information was unavailable when these previous life history studies were conducted, it was not possible to disentangle these potential cases of repeated *in situ* convergent evolution via natural selection from a pattern caused by evolution and subsequent dispersal amongst islands. For example, if skink populations on bird-only islands were not closely related, it would suggest that their phenotype evolved independently on each island, thereby suggesting convergent evolution due to natural selection pressure by bird predators. On the other hand, if all bird-only predator island skink populations shared a recent common ancestor exclusive of bird + snake island populations, then one could not distinguish the possibility of selection on life history and phenotype from a founder effect and subsequent dispersal to other islands.

We evaluate the diversity of phenotype and life history strategies of *P. latiscutatus* on the Izu Islands in a newly constructed phylogenetic framework. We use phylogenetic comparative methods to evaluate the relationships between a suite of life history characteristics and color pattern and link those results to an island’s predator assemblage. Finally, because the identity of island colonizers and their relative timing of colonization shape the evolution of community assemblages (*e.g.* [23]), it is important to uncover general biogeographic patterns in the island system. We therefore also compare the pattern of *P. latiscutatus* colonization of the Izu Islands with that of other taxa inhabiting the islands by consulting the latest knowledge on geological history and geochronology of island formation of the Izu-Bonin Island arc [25]–[27].

## Methods

### Study Islands, Life History and Phenotypic Data Collection

Life history traits and juvenile color pattern of *Plestiodon latiscutatus* (formerly *Eumeces okadae*; [6]) were studied in ten of the Izu Islands (Oshima, Toshima, Niijima, Shikine, Kozu, Tadanae, Miyake, Mikura, Hachijokojima and Aogashima), in the Izu Peninsula (Japanese mainland) and in the small offshore island (Hatsushima) off the east coast of the Izu Peninsula (Fig. 1). These islands, ranging in area from 10 to 9,908 ha, are all volcanic and located off the south coast of central Japan linearly from north (Oshima) to south (Aogashima) over the distance of ca. 230 km (Fig. 1). The climates, under the influence of the warm temperate water of the Kuroshio Current, are uniformly mild with average air temperatures of 16.2–17.9 C. Because of rich annual rainfall reaching 2000–3000 mm, the islands are well vegetated with broad-leaved evergreen forest dominated by *Castanopsis cuspidata* and *Machilus thunbergii* and secondary forest dominated by the deciduous trees *Alnus sieboldiana* and *Hydrangea macrophylla*. Terrestrial reptile and mammalian faunas of the Izu Islands and Izu Peninsula are summarized in Table 1. Predation regime (fauna and abundance), consequence of predatory mammal introduction, prey resource use and other ecologically relevant information can be found in the literature [9], [12].

Protocols, procedures and methods to obtain life history data both from the field and laboratory followed Hasegawa [9]. In brief, life history traits were based on data from intensive mark-recapture studies on Miyake from 1977 to 1984 [9], [21], and from less intensive mark-recapture studies conducted for the six other insular populations (Oshima, Toshima, Shikine, Kozu, Mikura, and Aogashima) from 1981 to 1984. The two Izu Peninsula populations (Daiyusan and Hiekawa), an offshore island (Hatsushima) and two Izu Islands (Niijima and Hachijokojima) were studied with mark-recapture methods from intermittently from 1994 to 2012. Snout-vent length (SVL) and body mass were measured to the nearest 1 mm and 0.1 g, respectively. The lizards were sexed, males were considered mature if exhibiting reddish nuptial (red or orange) coloration around the head. Maturity and reproductive conditions of females were determined [28]. In the spring (April–May), gravid (reproductive) females were classified as mature and reproductive in that year if they were either gravid or spent; otherwise they were classified as either immature or mature, but non-reproductive. Presence of mature but nonreproductive females was taken as evidence of missed reproductive opportunities. Clutch size was determined from the counts of yolked ovarian follicles, oviductal eggs, and corpora lutea in the female body cavities and from eggs in natural nests. At least 10 gravid females captured on each island during the late spring and early summer were brought back to the laboratory to lay eggs. Females were individually maintained in plastic containers with damp peat moss and small flat stones for a nesting site. Within 12 h of egg laying, the body masses of post-egg-laying (spent) females and of individual eggs were measured to the nearest mg. SVL, tail length, and body mass of hatchlings were measured to the nearest mm and mg within one day of hatching. Stripe pattern and blue tail coloration were individually scored for the hatchling lizards. The vividness of each of the head stripe and five body stripes was scored subjectively from absent (0), faint (1), obscure (2) and vivid (3) for dorsal, dorso-lateral and lateral stripes both in bead and body, and a sum of score for each stripe was calculated for each hatchling. Thus, sums of stripe scores varied from 0 for non-stripe to 18 for the most intensely striped individuals. For blue tail coloration, we measured the length of pure blue colored portion of tail (without the anterior stretch of black body stripes), and its proportion to the total length of tail was calculated for the individual lizards.

### DNA Collection and Sequencing

We sampled multiple individuals from each of the major Izu Islands and the small island of Tadanae and from three “mainland” populations including Atami, Izu Peninsula, and Hatsushima, a continental island population off the east coast of the Izu Peninsula (Fig. 1; Table 1). We also sampled two individuals of *P. japonicus* as outgroups as numerous phylogenetic studies have inferred it as the sister lineage to *P. latiscutatus* [29]–[31], [74].

DNA was isolated from tissue using Qiagen DNeasy columns. We amplified two mitochondrial genes (cyt *b* and ND1) using primers CB1 and CB6THR [32] for cyt *b*; newly developed primers ND1-LATF, 5′-CTC TCC CTA ATC ATG CAC CCA TTT TTC AC-3′ and ND1-LATR, 5′-TGA GCT CCT TAG TGC AGG TTC AGA TCC TG-3′ for ND1; and one nuclear gene (R35) using primers R35F and R35R [30] using standard polymerase chain reaction (PCR) techniques (95°C for 2 min followed by 40 cycles of 95°C for 30 s, 55°C for 30 s and 72°C for 60 s). PCR products were cleaned using ExoSap-IT (USB Corp. Ohio, USA). Purified templates were dye-labeled using BigDye (Applied Biosystems, California, USA) and sequenced on an ABI 3077 automated DNA sequencer (Applied Biosystems) using the same primers. Nucleotide sequences were examined and aligned by eye and an open reading frame for these genes were verified using MacClade v4.08 [33]. The sizes of the final data sets were 953 bp (cyt *b*), 957 bp (ND1), and 612 bp (R35) for the number of individuals listed in Table 2. R35 sequences with two or more polymorphic sites were phased into individual alleles using Bayesian inference with PHASE 2.1.1 [34], [35].

**Table 2:** Number of individuals used in the phylogenetic analyses.

	Number of individuals		
Locality	mtDNA	R35	R35 alleles
Mainland			
Atami	0	1	2
Izu Peninsula	14	16	26
Hatsushima	5	8	13
Izu Islands			
Oshima	14	15	15
Toshima	13	13	14
Niijima	14	13	22
Shikine	10	13	21
Kozu	14	13	21
Tadanae	14	9	15
Miyake	5	6	10
Mikura	15	14	15
Hachijojima	14	15	15
Hachijokojima	15	15	15
Aogashima	8	10	10
**Total = **	**155**	**161**	**214**

The mtDNA data set includes the combined cyt *b* and ND1 mtDNA genes. R35 nuclear alleles were inferred using PHASE 2.1.1.

### Time-calibrated Phylogenetic and Biogeographic Analyses

#### Mitochondrial DNA

Reconstructing colonization history requires knowledge of a taxon’s phylogenetic history and age of lineage divergences. Bayesian phylogenetic analyses assuming a relaxed molecular clock permit the simultaneous estimation of phylogeny, divergence time [36], [37], and biogeographic history [38] while also incorporating rate heterogeneity among lineages and phylogenetic uncertainty (and thus, estimates of error) in the tree estimation process. Moreover, these analyses estimate statistical support for phylogenetic and biogeographic reconstructions by calculating Bayesian posterior probabilities.

We used beast v1.7.3 [37] to estimate the phylogeny, divergence times, and biogeographic history using the combined mtDNA data set (cyt *b* and ND1) for the 155 sampled individuals of *P. latiscutatus*. Because assuming different nucleotide substitution models for individual data partitions improves both phylogenetic and divergence time estimation [31], [39], we calculated the best partitioning scheme and substitution models for the codon positions of each gene using Partitionfinder v1.0.1 [41]. Partitionfinder recommended three total partitions: cyt *b* codon position one, ND1 two; cyt *b* two, ND1 three; cyt *b* three, ND1 one and the substitution models TrN + G for partitions one and three, and HKY + G for partition two.

Estimating divergence times from molecular data requires some *a priori* estimate ages for at least one divergence. These are commonly estimated by incorporating fossil taxa as age constraints to “calibrate” the relaxed molecular clock. However, there are no known *Plestiodon* fossils that can be used as calibration age constraints. We therefore used the age distribution of the most recent common ancestor of *P. japonicus* and *P. latiscutatus* inferred by a multi-locus time-calibrated phylogenetic analysis of *Plestiodon* [30] as our age calibration constraint. Although secondary calibrations have been rightly criticized for potentially compounding date estimation error [40], we note that Bayesian age estimation permits the explicit incorporation of this error by permitting age calibration constraints (rather than point estimates) in the form of statistical distributions, thus eliminating at least one negative feature of secondary calibrations [31]. Also, the age distribution is broad and thus likely not overly precise (see Results).

Simultaneously with estimating phylogeny and divergence times, we inferred ancestral biogeographic area using the discrete traits model of Lemey et al. [38]. We coded all *P. latiscutatus* individuals into groups representing each island or mainland peninsula populations.

Each BEAST analysis was run for 10^7^ generations and sampled every 2000 generations. We modeled the age of the root of the tree (*P. japonicas+P. latiscutatus*) as a normal distribution of ages with a mean = 6.3 Ma and standard deviation = 1.38 (95% CI = 3.6–9.0 Ma; [31]) and enforced a separate lognormal relaxed molecular clock for the cyt *b* and ND1 data. We otherwise used default priors except that we modeled the mean rate of the cyt *b* and ND1 molecular clocks at uniform distributions with bounds of 0.0 and 0.1 substitutions per site. We ran eight BEAST analyses assuming a birth-death tree prior. To determine convergence amongst each analysis, we constructed cumulative posterior probability plots for each clade using the *cumulative* function in AWTY [42]. Stationarity was assumed when the cumulative posterior probabilities of all clades stabilized. If posterior probability estimates for clades were similar in the analyses, the results were combined. We interpret posterior probabilities ≥0.95 as suitably strong support for both phylogenetic reconstruction and estimation of ancestral biogeographic area [43].

#### R35 nuclear locus analyses

Preliminary phylogenetic analyses of the R35 alleles revealed very little phylogenetic structure due to very few nucleotide substitutions (only six parsimony-informative sites), and therefore imprecise information about the evolutionary history of the sampled *P. latiscutatus* populations. We instead visualized the genetic diversity of R35 amongst the sampled localities by calculating the allele frequencies for each population and visually inspected them for general trends.

### Comparative Analyses of Life History Traits

We performed phylogenetic comparative analyses to estimate correlations amongst life history characteristics (correlations of phylogenetic independent contrasts) and then linked those life history traits to the type of predators on multiple Izu Islands (phylogenetic ANOVA). For statistical analysis, arithmetic means of the sampled life history and phenotype characters were converted to independent contrasts to remove non-independence due to phylogenetic history [44] under different phylogenetic scenarios (see below). Correlations of independent contrasts of life history traits were performed using the CAPER package in R [45] using scripts written by M.C.B. We conducted phylogenetic generalized least squares (PGLS) analysis of variance (phylogenetic ANOVA) analyses [46] to determine how variation in life history traits correlates to the presence of predators on each island after accounting for phylogenetic relationships. There exist three major classes of skink predators on the Izu Islands including weasels, birds, and snakes, but we focus only on the potential influence of snake and bird predation on life history variation. Unlike weasels that are historically native to Oshima (they were subsequently introduced into Toshima in the 1930s, Hachijojima in the 1960s, Miyake and Aogashima in the 1980s), and predatory birds that inhabit all islands, snakes inhabit eight of the 11 islands sampled for this study and likely derive from independent colonizations [19]. Therefore, snakes offer multiple independent opportunities to assess the affects on the presence of different predators on the life history traits of their prey, *P. latiscutatus*. Moreover, snake-less islands offer an opportunity to assess the effects of bird-only predation on *P. latiscutatus* life history and color evolution.

Phylogenetic ANOVA (pANOVA hereafter) analyses were performed using the GEIGER [47] package in R and scripts written by M.C.B. We regressed the mean values of both hatchling and maternal life history traits against a dummy variable coded 0 or 1 (0 = island has snakes; 1 = island does not have snakes). The overall correlation coefficient (R) represents deviations from the mean of the comparison group and was tested for significance using a t-test [46].

**Figure 2:**
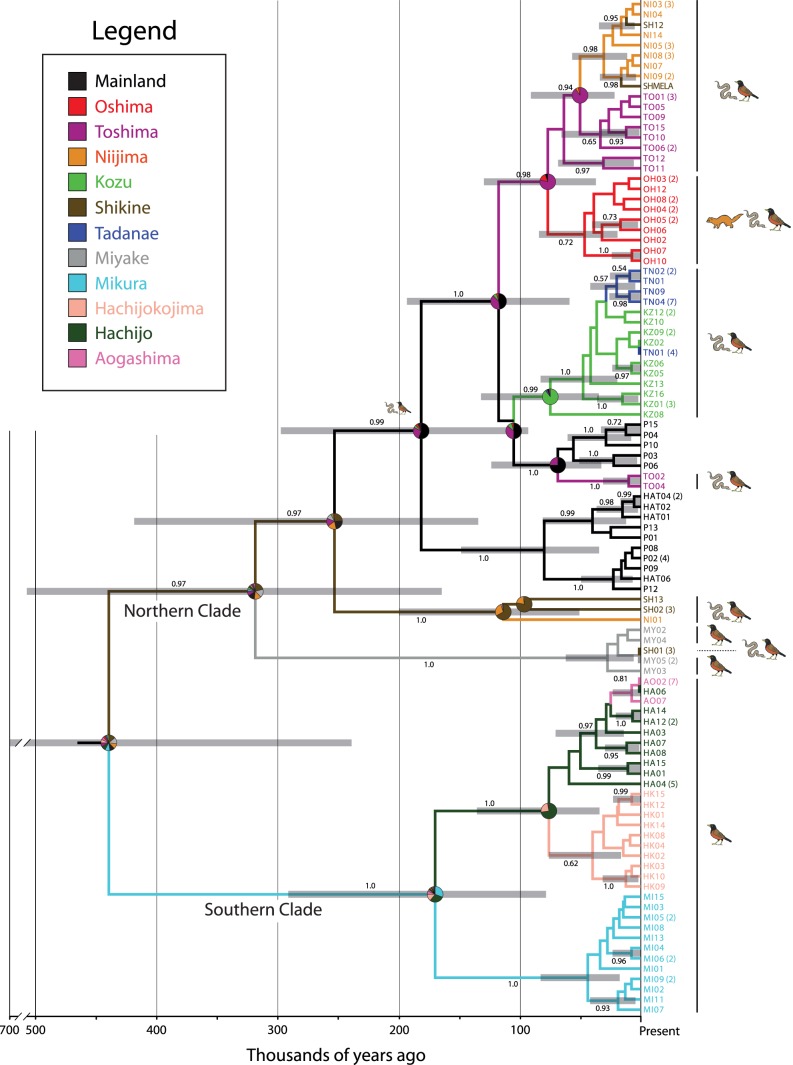
Time-calibrated intraspecific phylogeny of *Plestiodon latiscutatus*. Colors refer to separate islands and to island colors used in Fig. 1. Numbers after sample names indicate the number of sampled individuals that share that haplotype. Values above or below the nodes indicate Bayesian posterior probabilities calculated from 8000 trees in the posterior distribution. Grey bars indicate the 95% credible set of ages. Pie charts indicate the relative probability that the ancestral population of a clade inhabited specific Izu Islands indicated by each color.

Because both independent contrasts and phylogenetic ANOVA analyses use phylogenetic information to remove the effect of non-independence caused by the organisms’ shared evolutionary history, the results are therefore fundamentally reliant on the underlying phylogeny. Inspection of the mtDNA phylogeny (Fig. 2) reveals multiple populations that are not monophyletic including the mainland, Miyake, Niijima, Shikine, and Toshima populations. This pattern could be indicative of multiple colonizations to these islands (i.e., the pattern represents the true colonization history), or it could result simply from a stochastic process where drift has not eliminated older mtDNA haplotypes such as incomplete lineage sorting (i.e., the pattern is an artifact of molecular evolution). Incomplete lineage sorting is a phenomenon where ancestral alleles (or haplotypes, in the case of mtDNA) that are present before a lineage splits (i.e., when two or more populations are reproductively isolated) are retained in its descendant lineages after the divergence [48]. These ancestral alleles will be lost to genetic drift over time and replaced by new alleles unique to the new descendant lineages; however, during short time frames, there is a chance that these alleles will not be lost due to drift (e.g., [49]–[51]). The distinction between these two processes (multiple colonizations and incomplete lineage sorting) has significant effects on how we interpret life history evolution. We assume that our sampled life history data represents the frequency of these traits on each island (e.g., the mean hatchling SVL for the entire population of Aogashima is 30.4±1.3 mm; Table 1). If two or more lineages colonized an island, then it would suggest that each lineage convergently evolved the same distributions of life history traits, thereby increasing the strength of correlations amongst traits in subsequent comparative analysis. This is not problematic if indeed the islands were colonized multiple times. However, if the non-monophyly of island populations is simply an artifact of molecular evolution, and that each island was indeed colonized only once, then assuming multiple colonizations would create false positive support for our trait correlation analyses.

We therefore performed the independent contrast and pANOVA comparative analyses, assuming both the multiple colonization scenario supported by our inferred phylogeny, and single colonization scenarios that assume the presence of two distinct lineages on an island is an artifact of incomplete lineage sorting. For the multiple colonization scenario, we performed the comparative analyses using a phylogeny pruned to the minimum number of possible independent colonizations. Assuming a single colonization to each island poses a challenge because there are multiple possible resolutions of our phylogeny compatible with a single colonization scenario (e.g., Toshima may be sister to a mainland or Niijima + Shikine clade [Fig. 2]). We therefore developed a novel method that accounts for the multiple possible resolutions whereby we sample a tree from the posterior distribution of trees estimated by the BEAST analysis, and prune the tree to include only one randomly chosen individual per island. We then use this tree for the IC and pANOVA analyses. We repeated this process for all 8000 trees in the posterior distribution thereby creating a distribution of p values from each test using R scripts written by M.C.B. Because there is no objective way to interpret distributions of p values, we subjectively interpret the results assuming that a distribution of p values that cluster closer to 0 are more suggestive of a statistical relationship between two variables than a distribution that includes mid- to high p values. We note that the tree topology of the pruned tree will depend on which of the multiple island lineages are retained (i.e., sampling any one of the Toshima lineages would change the topology of a single tree). However, because this process is random across the 8000 trees, we assume there is no systematic bias. Indeed, repeating the analysis with different random number seeds yields identical results (not shown). We also emphasize that this method also incorporates phylogenetic uncertainty as not all clades have a posterior probability of 1.0. Life history data, DNA data, and R scripts are available from the Dryad Digital Repository: doi:10.5061/dryad.v47s1.

### Ethics Statement

Animals were captured by hand or by a fishing pole with a mealworm attached to the end of a string. When euthanasia was necessary, it was performed via overdose of sodium pentobarbital. All animal work was reviewed and approved by the Toho University Bioethics and Animal Ethics Committee (approval number: #12–51–242). Fieldwork and sample collection on Kozu was approved by the village of Kozu on 1 July 2011 (no permit number issued) in accordance with their ordinance governing plant and animal protection. No specific permission is required for fieldwork or collection on the remaining Izu Islands. This study did not involve endangered or protected species.

## Results

### Phylogeny and Biogeographic Reconstructions

#### MtDNA

The Bayesian analyses achieved stationarity by two million generations. The consensus of 8000 trees in the posterior distribution, clade posterior probabilities, 95% credible intervals of age, and biogeographic reconstructions are provided in Fig. 2. The most notable broad pattern of *P. latiscutatus* colonization of the Izu Islands is a basal split in the phylogeny between a northern clade including populations from the Izu Peninsula, Miyake, and islands north, and a southern clade containing Mikura and islands south. The northern clade comprises two clades: a clade inhabiting Miyake (and a Shikine haplotype), and a clade containing populations from the Izu Peninsula and the northern Izu Islands (Fig. 2). The populations inhabiting Niijima, Shikine, and Toshima are not monophyletic. Furthermore, Niijima and Shikine are inhabited by two distantly related clades. The southern clade is composed of clades inhabiting Mikura, Hachijokojima, and Hachijojima, with the Aogashima population nested deep within the Hachijojima clade.

Because biogeographic reconstructions based on our data are largely equivocal (see Fig. 2), it is not possible to reconstruct the step-by-step pattern of colonization history of *P. latiscutatus*. An exception is the clade containing Toshima, Niijima, and Shikine populations; the latter two dispersed from Toshima or all three once shared a landmass. The population inhabiting Tadanae derived from a source population in the geographically proximate Kozu. Finally, Aogashima was very recently colonized by dispersal from Hachijojima. A striking pattern is that the crown age for most island clades is less than 100,000 years, many less than 50,000 years.

#### R35

**Figure 3:**
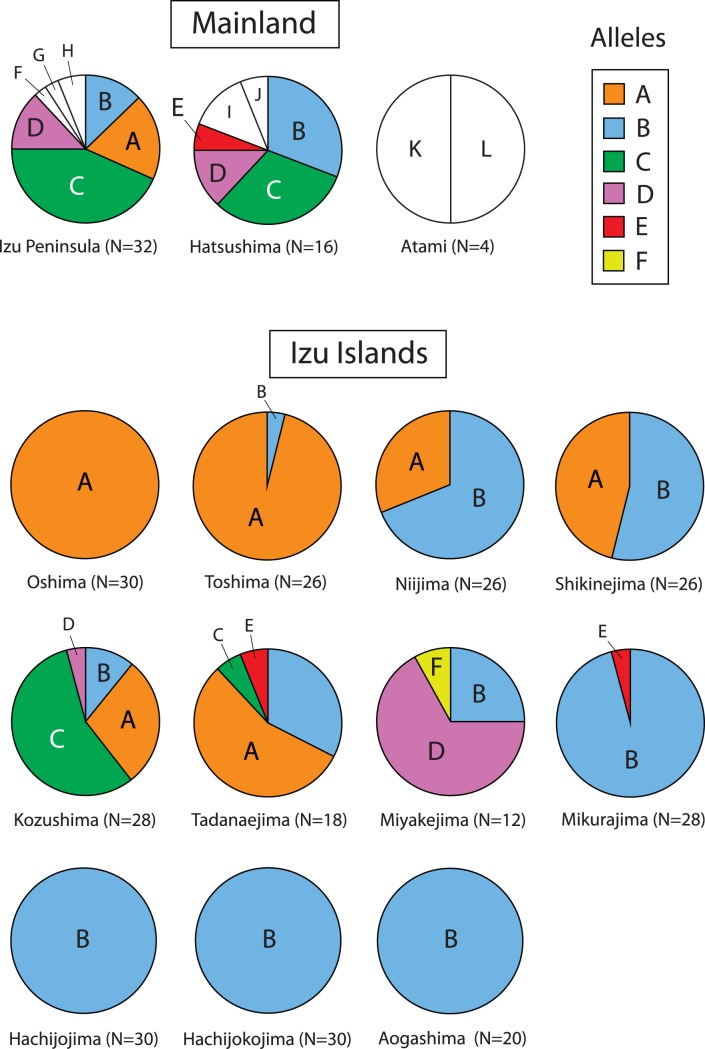
Pie charts representing the relative proportion of phased R35 allele alleles in the mainland and Izu Islands. The order of presentation of the pie charts is the same as the order of the islands north to south (see Fig. 1).

A total of 12 alleles are present amongst the sampled *P. latiscutatus* populations (Fig. 3). Two alleles, A and B, are the most geographically widespread, being present in 12 and seven of the 14 populations. The mainland populations possess the most allelic diversity with seven and six alleles in the Izu Peninsula and Hatsushima populations. The B allele, although present in most populations, is the dominant allele in the southern mtDNA Izu Islands clade (Mikura, Hachijojima, Hachijokojima, and Aogashima; Fig. 1), whereas the A allele is more common in the northern Izu Islands clade. Kozu and Tadanae are unique in that they posses the most number of different alleles in the Izu Islands populations (four each) and having the presence of the C (both populations, the dominant allele in Kozu), D (Kozu), and E (Tadanae ) alleles. Miyake is also notable for the absence of the B allele, presence of the F allele, and dominance of the D allele.

### Phylogenetic Comparative Analyses

**Figure 4:**
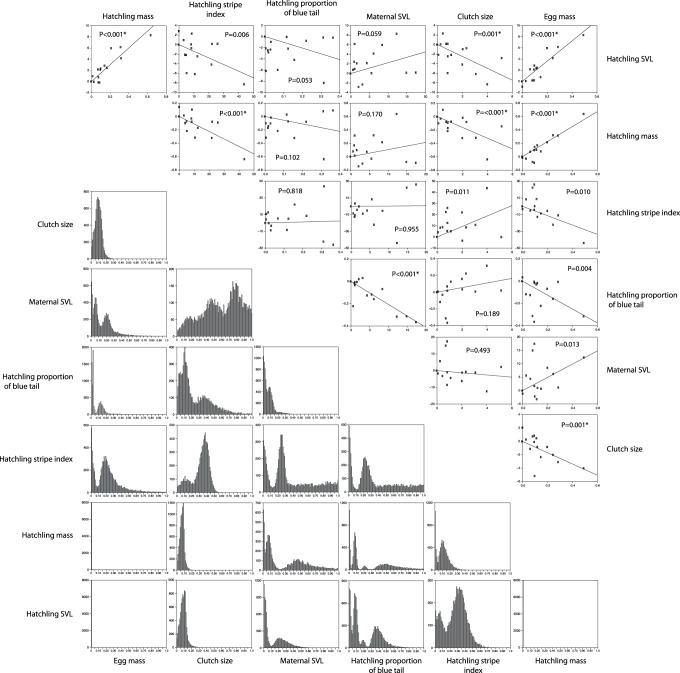
Results of the regressions of independent contrasts of life history and phenotypic traits. P-values that are significant after table-wide sequential Bonferroni-correction are indicated by an asterisk and are identical to those in Table 3. Graphs above the diagonal are regressions assuming that Niijima, Shikine, and Toshima were colonized multiple times as suggested by phylogenetic analysis (Fig. 2). Graphs below the diagonal are distributions of p values of regressions of life history and phenotypic traits calculated from 8000 trees in the posterior distribution and assuming that each island was colonized once and that the presence of multiple lineages on an island is due to artifacts of molecular evolution including incomplete lineage sorting. Values shifted towards zero indicate subjective support for the relationship of the two variables.

The results of the regressions of phylogenetically independent contrasts are shown in Fig. 3 and Table 3. Multiple significant correlations (after sequential Bonferroni correction) make intuitive sense including significant positive correlations between hatchling mass and SVL and negative correlation between clutch size and egg size. However, there are less intuitive correlations including an inverse correlation between hatchling mass and stripe vividness, and a higher proportion of blue in the tail correlated with decreasing egg mass and smaller maternal SVL. The statistically significant correlations in the multiple colonization scenario also have p-value distributions shifted towards zero (typically less than 0.20; Fig. 4).

**Table 3:** Results of regressions of independent contrasts of life history and phenotype variables assuming that the islands of Niijima, Shikine, and Toshima were colonized more than once (as indicated by the phylogeny in Fig. 2).

Independent variable	Dependent variable	T	R^2^	P
Hatchling SVL	Hatchling mass	11.16	0.90	<0.001*
Hatchling SVL	Hatchling stripe vividness	−3.21	0.42	**0.006**
Hatchling SVL	Hatchling proportion of tail is blue	−2.12	0.24	0.053
Hatchling SVL	Maternal SVL	2.06	0.23	0.059
Hatchling SVL	Clutch size	−4.34	0.57	0.001*
Hatchling SVL	Egg mass	11.43	0.90	<0.001*
Hatchling mass	Hatchling stripe vividness	−5.61	0.69	<0.001*
Hatchling mass	Hatchling proportion of tail is blue	−1.75	0.18	0.102
Hatchling mass	Maternal SVL	1.45	0.13	0.170
Hatchling mass	Clutch size	−4.62	0.60	<0.001*
Hatchling mass	Egg mass	9.86	0.87	<0.001*
Hatchling stripe vividness	Hatchling proportion of tail is blue	0.23	0.00	0.818
Hatchling stripe vividness	Maternal SVL	0.06	0.00	0.955
Hatchling stripe vividness	Clutch size	2.91	0.38	**0.011**
Hatchling stripe vividness	Egg mass	−3.00	0.39	**0.010**
Hatchling proportion of tail is blue	Maternal SVL	−8.26	0.83	<0.001*
Hatchling proportion of tail is blue	Clutch size	1.38	0.12	0.189
Hatchling proportion of tail is blue	Egg mass	−3.46	0.46	**0.004**
Maternal SVL	Clutch size	−0.70	0.03	0.493
Maternal SVL	Egg mass	2.86	0.37	**0.013**
Clutch size	Egg mass	−3.94	0.53	0.001*

Positive or negative values of T indicate that the relationships are positive or negative, respectively. P-values ≤0.05 are in bold, and P-values that are significant after table-wide sequential Bonferroni-correction are in indicated with an asterisk.

**Figure 5:**
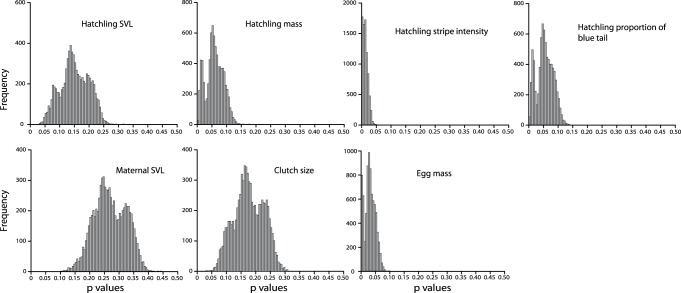
Results of the phylogenetic ANOVA analyses testing the correlation between life history and phenotypic variation to the presence of snake predators on each island calculated from 8000 trees in the posterior distribution and assuming that each island was colonized once. Values shifted towards zero indicate subjective support for the relationship of the two variables.

The pANOVA analyses assuming a multiple colonization scenario found that low hatchling mass, low egg mass, and increased stripe vividness are significantly correlated with the presence of snake predators (Table 4). In addition, increased proportion of blue in the tail is also positively associated with the presence of snakes, but this is not statistically significant after a sequential Bonferroni correction (P = 0.041). The single colonization scenario analyses also infer highly suggestive evidence for these same variables. This is especially the case with stripe vividness as all p-values estimated from 8000 pruned trees from the posterior distribution are <0.05 (Fig. 5). Although we cannot properly assess statistical significance with the single colonization scenarios given that they are distributions of p-values (with the possible exception of those variables in which all 8000 analyses had p-values <0.05), the results of both the IC (Fig. 4) and pANOVA (Fig. 5) for both the multiple colonization scenarios broadly support the same conclusions as the single colonization analyses, and therefore our results are robust even if multi-lineage islands are an artifact of incomplete lineage sorting.

**Table 4:** Results of the phylogenetic ANOVA analyses testing whether variation in life history and phenotype traits are associated with the presence of snake predators on each island.

Trait	P
Hatchling SVL	0.072
Hatchling mass	0.008*
Hatchling stripe vividness	<0.001*
Hatchling proportion of blue tail	**0.041**
Maternal SVL	0.249
Clutch size	0.072
Egg mass	0.006*

This analysis assumes that the islands of Niijima, Shikine, and Toshima were colonized more than once. P-values ≤0.05 are in bold, and P-values that are significant after table-wide sequential Bonferroni-correction are in indicated with an asterisk.

## Discussion

Because the Izu Islands are climatically and floristically similar, and differ primarily in the diversity of vertebrate predators, this island system offers a rare opportunity to analyze repeated convergent evolution of life history traits and color pattern across similar habitats, but under conditions where predator diversity may be the primary driver of phenotypic and life history evolution. Below, we discuss the results of our phylogenetic comparative analyses and the relation of life history and color pattern diversity in the lizard *Plestiodon latiscutatus* to an island’s predator assemblage. Because of small sample size, we are unable to discuss life history and color pattern data for the Izu Peninsula and Hachijojima population in detail. We evaluate the time-calibrated colonization history of *P. latiscutatus* with reference to the latest geochronological data for tectonic events and geologic ages of island formation [25], [27]. Then we compare these results to the other studied plants and animals (including its primary snake predator, *Elaphe quadrivirgata*) to evaluate general patterns of colonization and assembly processes of insular ecological communities [23].

We note that our strongly supported phylogeny is nonetheless based on a single locus (mtDNA), and it is probable that the mtDNA history does not exactly match the population history of *P. latiscutatus*. On the other hand, we would expect any phylogenetic error to manifest among recent populations (assuming that drift is more likely to eliminate older haplotypes). Because the bird predator island clades reside in separate clades at the root of the phylogeny supported by PPs >0.95 (Fig. 2), it is therefore unlikely that the bird predator islands actually form a clade exclusive of other populations. We also note that recent divergences may be systematically overestimated when external molecular age calibrations are used [52], [53]. However, if the ages of *P. latiscutatus* clades are younger, it would make the rapid phenotypic evolution in the species even more remarkable.

### Predator Driven Convergent Evolution of Life History and Color Pattern

Using statistical analyses that account for the effect of shared evolutionary history, we support many of the conclusions of Hasegawa [9], [12]. We find statistically significant support for correlations between many life history and phenotype variables (Table 3, Fig. 4). More importantly, variation in hatchling mass and stripe vividness is significantly associated with the presence of snake predators, and inversely associated with bird-only predation (Table 4; Fig. 5). Similarly, our phylogenetic tree (Fig. 2) demonstrates that one or both correlated life history character suites evolved independently at least twice. Populations with the bird-only predator phenotypes (large hatchlings, low stripe index) inhabit both the Northern (Miyake) and Southern (Hachijokojima, Aogashima) clades. Similarly, populations with phenotypes associated with snake-only predation (small hatchling size, high stripe index, blue tail) inhabit both a majority of the Northern clade and Mikura in the Southern clade.

By and large, our single colonization scenario analyses (which also incorporate phylogenetic uncertainty) subjectively support the conclusions of the independent contrast and pANOVA analyses assuming multiple colonizations. Although there is no statistically satisfying way to interpret distributions of p values inferred by these analyses, we note that insignificant IC and pANOVA tests in the multiple colonization analyses (Figs. 3 and 4) have very wide distributions of p values, whereas significant multiple colonization results have distributions of p values shifted towards zero, typically <0.20. Regardless, that both the single and multiple colonization analyses yield similar results demonstrates that the significant association of suites of life history and phenotype characters are is not due to different colonization scenarios and/or incomplete lineage sorting.

Based upon this, and previous studies, we classify populations of *Plestiodon latiscutatus* on the Izu Islands into three groups based on the abundances of *P. latiscutatus* and on the potential avian, snake, and mammalian predators [9], [12]. The first group is bird only islands including populations on Aogashima, Hachijokojima and Miyake free from predation by snakes and carnivorous mammals. The lack of these “top” predators is likely responsible for the high abundance of *P. latiscutatus* and insectivorous birds. Of these insectivorous birds, *Turdus celaenops*, a predator upon juvenile lizards [20], was particularly abundant on these bird-only islands [11], [12]. The second group is bird + snake islands of Mikura, Kozu, Niijima, Shikine and Toshima, which lack only mammalian predators. The bird + snake islands supported moderately high densities of *P. latiscutatus* and the snake *E. quadrivirgata* [7] and low densities of avian predators. The third group inhabits a single island (Oshima) with the richest predator fauna, including the native mammalian predator, the Japanese weasel (*Mustela itatsi*). The density of *P. latiscutatus* Oshima was lowest among the islands. This inverse correlation between predator richness and lizard density found on the Izu Islands is a typical example of the worldwide pattern of relationship between predation impact and density of insular lizards [54]. However, very few studies have demonstrated a clear relationship between patterns of predator community structure and lizard life histories in insular environments (e.g., [55]).

Our previous research documented that the *P. latiscutatus* hatchlings were larger and more drab-colored on islands with high bird predation and no snake predators [9], [12]. Hasagawa [9], [12] surmised that the small size of the primary bird predator, the Izu Islands Thrush (*Turdus celaenops*), limits the body size of prey it can consume [20] and thus there was strong selective pressure to favor large hatchling SVL and mass, and therefore large eggs and low clutch sizes. Moreover, given the high visual acuity of bird predators [56], natural selection would favor an inconspicuous, cryptic color pattern. *T. celaenops,* a gape-limited insectivorous bird, attacks juvenile *P. latiscutatus* by attacking from a perch above the juvenile lizards basking or foraging on the brown colored leaf litter [20]; therefore, the striped and blue colored tail of juvenile *Plestiodon* lizard would be conspicuous to these predators. Indeed, field experiments using clay lizard models demonstrated that birds will preferentially attack portions of clay lizard models conspicuously colored blue [57]. It is thereby reasonable to conclude that avian predation on juvenile lizards favor inconspicuous dull coloration in juvenile lizards.

Conversely, *P. latiscutatus* that inhabit islands with snake predators that can consume both adult and juveniles tend to produce smaller hatchlings, smaller eggs, larger clutches, and have conspicuous yellow stripes and blue tail. Contrary to the birds that attack from above, the snake *Elaphe quadrivirgata* stalks and attacks lizards, usually from behind, within short distance of ∼20–30 cm, and laboratory experiments using snake predator demonstrated that the snake struck the body of lizards with blue-painted tails less frequently than that of lizards with darkly painted tails [58]. The striped body trunk would be effective to disrupt both the snake’s perception of the lizard body and snake attacks at close range [59]. A combination of striped pattern and blue-colored tail therefore distract the predator’s attention away from the more vulnerable parts of the body (head and trunk), enabling lizards to escape from predation at the cost of losing their tails [58]–[61].

Other studies of insular lizard populations that focused on the combined effects of predation and intra-specific competition [62]–[68] reported similar causal relationships between life history traits and predation regimes. In other system with both bird and snake predators, dwarf chameleons did not adjust the color of their bodies when presented with either predator [69]. However, the chameleons increased the brightness of their colors when confronted with a snake. Although not directly analogous to our results, this chameleon study demonstrates that different aspects of chromatic (color) or achromatic (brightness) contrast are adaptations to the different visual systems of snakes and birds. Meta-analyses of island populations detected a broad pattern of correlated ecological and life history shifts toward increased population density and infrequent production of few, large offspring, termed the “island syndrome” [70]. Alternatively, specific environmental factors such as erratic environmental conditions or high predation pressure have been found to that reduce lizard population density and mold life history traits of insular lizard populations in the opposite direction, a phenomenon termed “reversed island syndrome” [66], [67].

In conclusion, in the Izu Island system, predation directly impacts upon the demographic parameters of lizard populations (toward either low or high density) and indirectly upon prey availability via intra-specific competition under different population densities [9], [12], [21]. Both the typical island syndrome (increased population density, infrequent production of few, large offspring) and reversed island syndrome (decreased population density, frequent production of many, small offspring) occur in the Izu Islands populations of *Plestiodon latiscutatus* due mainly to differential colonization history of bird, snake and mammal predators.

### Phylogeography of *Plestiodon latiscutatus* in Relation to Geochronology of the Izu Islands

In addition to allowing us to assess convergence in life history and juvenile color pattern phenotypes, our inference of *Plestiodon latiscutatus* phylogeny also informs the historical biogeography of the species. Although the biogeographic reconstructions at the root of the phylogeny are equivocal, likely due to the monophyly of most island populations with few island-specific phylogenetic grades, visual inspection clearly supports the hypothesis that extant mainland Izu Peninsula populations derive from ancestors that inhabited the Izu Islands. Although the biogeographic reconstructions are largely ambiguous in all but shallow nodes, there are nonetheless clear examples of recent dispersal amongst geographically proximate islands including the colonization of Aogashima from Hachijojima and Tadanae from Kozu. There are also multiple instances of putative dispersal between Niijima and Shikine, islands separated by ∼3 km, and from Shikine to Miyake.

All of the Izu Islands, and most of the Izu Peninsula and central Japan, are volcanic, formed in the complex interface of the Eurasian, Pacific, and Philippine plates. The Izu Islands are situated in a zone where the blocks of the Izu–Bonin arc’s upper crust have been successively accreted onto the Honshu arc for the past 15 million years [71], [72]. The collision and accretion of older island blocks triggered the formation of new volcanic islands behind the collision front thereby creating a series of Izu proto-islands. The present-day Izu Peninsula formed from a collision of Izu proto-islands with the main island of Honshu 0.5–0.7 Ma [26], and this collision may have triggered the formation of most of the present-day Izu Islands. Indeed, geochronology data indicates that at least Kozu and Niijima islands are younger (0.88–0.93 Ma) than the Proto-Izu block (1.7–7.4 Ma). It is therefore reasonable to assume that the volcanic activity that formed the current Izu Islands was present in the past and that islands (although not necessarily the present-day islands) have been continually present in the region for millions of years.

The results of our phylogenetic analyses in this geological context support an intriguing biogeographic hypothesis by Okamoto et al. [73] in their analysis of mainland *P. latiscutatus* phylogeography. In this biogeographic scenario, the common ancestor of *P. latiscutatus* and *P. japonicus* inhabited mainland Japan prior to the formation of the Izu Peninsula. The ancestor of the present-day *P. latiscutatus* lineage dispersed to the Izu proto-islands between 3 and 7.6 Ma. This date is coincident with the age of the split between *P. latiscutatus* and *P. japonicus* [31] and the formation of the Proto Izu Island block 1.7–7.4 Ma [27]. The *P. latiscutatus* lineage diversified on the Izu proto-islands and reinvaded the mainland after the formation of the Izu Peninsula [73]). Throughout this period, the ancestors of the extant island populations would have colonized these newly formed islands. Since 1 Ma, these islands included some of the present-day Izu Islands.

Okamoto et al.’s [72] Izu proto-island hypothesis is thus congruent with the ages of *P. latiscutatus* clades estimated by our phylogenetic analyses. If mainland Japan was colonized by the collision of *P. latiscutatus* inhabited Izu proto-islands, we would expect the age of the mainland Izu Peninsula lineages to coincide with this collision occurring 0.5–0.7 Ma (assuming limited or no dispersal from the proto-islands to the mainland prior to the collision). Our estimated age of the split between the clade containing the Izu Peninsula and most northern island populations is ∼0.7–0.24 Ma. Unfortunately, without fossil data, our estimates of divergence age may not become more precise, even with additional DNA data.

Another possibility is that the ages of the extant *P. latiscutatus* populations do not coincide with age of colonization because of processes of historical diversification. A striking contrast is the age of the *P. latiscutatus* lineage (i.e., the age of the split between that species and *P. japonicus*), approximately 3–7.6 Ma [31], and the relatively young age of the crown clade of extant populations (0.24–0.7 Ma). One potential explanation for this phenomenon is that this clade experiences high lineage turnover, a phenomenon in which birth and death of lineages is high (e.g., [74], [75]), resulting in an apparent lack of older lineages. That the mean ages of the clades inhabiting the modern Izu Islands are almost all less than 0.1 Ma also deserves explanation. This pattern too could be explained by high lineage turnover resulting in the extinction of older *P. latiscutatus* lineages. Another potential explanation is that the modern Izu Islands were formed, or became isolated, in the past 100,000 years, however, the ages of the modern Izu Islands except Kozu and Niijima (0.88–0.93 Ma, respectively; [27]) are not known with certainty.

The phylogenetic split between islands north of Mikura (including the mainland) and south of Miyake is unique when compared to previous Izu Islands phylogeographic studies. This pattern is also seen in the distribution of R35 alleles. The northernmost island, Oshima, contains a single allele (A) that also dominates the Toshima population immediately south. The A allele continues to be present in the islands as far southern as Kozu, but at a lower frequency. The A allele is completely absent from the South Clade, and is replaced by the B allele in the three southernmost islands and all but one individual in Mikura. This north-south split result is puzzling since Miyake and Mikura are geographically close (∼17 km), and therefore we would expect some dispersal. This pattern could be explained by an ancient separation of populations prior to the collision of the Izu proto-island with the mainland although the age of the north-south split (0.24–0.7 Ma) is younger than or roughly simultaneous with the hypothesized time of this collision (0.5–0.7 Ma).

A striking finding is that the age of the populations on Miyake is very young (0.06–0.5 Ma), yet the entire lineage (which also includes an individual from Shikine) is much older (0.16–0.56 Ma) – a pattern similar to the Bermuda skink, *Plestiodon longirostris* [30]. Geologic evidence suggests that Miyake may have formed approximately 10,000 years ago (it is presently an active volcano) [76]. Thus, if the bird predation-mediated drab coloration and life history characteristics evolved *in situ* on Miyake, then it must have evolved very quickly.

Miyake is also notable for the dominance of the R35 D allele that is only found in one A/D heterozygote in Kozu, but more common on the Izu Peninsula. We lack the data to fully explain this phenomenon, but we note that, since the introduction of the Japanese weasel (*Mustela itatsi*) in the early 1980s, the population size of Miyake *P. latiscutatus* has undergone a massive decline to one-thousandth to ten thousandth of its pre-1980s population size [8]–[10]. It is quite likely that this bottleneck has severely affected the historical frequencies of R35 alleles.

The phylogeography of *P. latiscutatus* is unique when compared to other Izu Islands taxa studied to date. The most relevant comparison is to four-lined ratsnake, *Elaphe quadrivirgata*, the primary snake predator of the Izu Islands *P. latiscutatus*. Kuriyama et al. [19] found that all Izu Islands populations descend from mainland ancestors within the past 0.20–0.58 Ma, and the intraspecific phylogeny of *E. quadrivirgata* is composed of a clade inhabiting Izu Peninsula, Oshima, and Mikura populations, and another clade containing Toshima, Niijima, Shikine, Kozu, and Tadanae populations. The latter pattern is similar to that seen in *P. latiscutatus* and is likely due to the close proximity the islands (Fig. 1) and potential land connections during Pleistocene glacial maxima. However, there exist multiple dispersals of *E. quadrivirgata* to and/or from the mainland and Oshima, and this pattern strongly contrasts with *P. latiscutatus* where the Oshima population is monophyletic and therefore descended from a single colonization.

The phylogeographic pattern of *P. latiscutatus* is similar to multiple patterns inferred for *Apodemus* mice in that there is a close relationship between Niijima, Shikine, and Miyake populations, but differ in that Oshima and Kozu were separately colonized by a lineage of mainland *Apodemus* mice [16]–[18]. It is uncertain, however, that the Izu Islands populations of *Apodemus* mice showed deep split from mainland population like *P. latiscutatus*. Finally, our results differ from phylogeographic studies of *Campanula* plants [13], [14] and *Euhadra* snails [15], which found that the entire Izu Islands was colonized once. Taken together, these results demonstrate that colonization of the Izu Islands did not follow a general pattern, and instead the islands’ communities experienced different patterns of colonization and therefore offer great potential to explore replicated patterns and processes of community assembly.

## Conclusions

The significant associations among predator fauna, lizard population density, and life history traits suggests that predator identity and abundance have a direct role in molding the observed geographical distribution of life-history traits in the Izu Islands *Plestiodon latiscutatus* [12]. As far as we know, the Izu Islands *P. latiscutatus* system is the most comprehensively examined classic example of the island syndrome in lizard populations, with datasets for predation regime, lizard density, intensity of intraspecific competition for prey resources, intensity of male-male competition for gravid females, degree of female choice, and complete datasets of life history traits [8], [9], [12], [20]–[22]. To fully develop a mechanistic understanding of the proximate factors responsible for evolutionary shifts of life history and other traits in relation to ecological factors requires further analysis of the potential causative physiological, endocrinological, immunological, neurological and behavioral mechanisms controlling particular life history traits (e.g., [65]). Nonetheless, our phylogenetic approach to studying independent and repeated evolution is an important step for the future mechanistic understanding of phenotypic evolution.
